# Long-term Results after CT-Guided Percutaneous Ethanol Ablation for the Treatment of Hyperfunctioning Adrenal Disorders

**DOI:** 10.6061/clinics/2016(10)08

**Published:** 2016-10

**Authors:** Nathan Elie Frenk, Fernando Sebastianes, Antonio Marcondes Lerario, Maria Candida Barisson Villares Fragoso, Berenice Bilharinho Mendonca, Marcos Roberto de Menezes

**Affiliations:** I Hospital das Clínicas da Faculdade de Medicina da Universidade de São Paulo, Instituto de Radiologia, Serviço de Intervenção Guiada por Imagem, São Paulo/SP, Brazil; II Faculdade de Medicina da Universidade de São Paulo, Disciplina de Endocrinologia, Departamento de Medicina Interna, São Paulo/SP, Brazil; III Instituto do Câncer do Estado de São Paulo, Serviço de Radiologia e Intervenção Guiada por Imagem, São Paulo/SP, Brazil

**Keywords:** Ablation Techniques, Adrenocortical Adenoma, Pheochromocytoma, ACTH-independent Macronodular Adrenal Hyperplasia, Cushing Disease

## Abstract

**OBJECTIVES:**

To evaluate the safety and long-term efficacy of computed tomography-guided percutaneous ethanol ablation for benign primary and secondary hyperfunctioning adrenal disorders.

**METHOD:**

We retrospectively evaluated the long-term results of nine patients treated with computed tomography-guided percutaneous ethanol ablation: eight subjects who presented with primary adrenal disorders, such as pheochromocytoma, primary macronodular adrenal hyperplasia and aldosterone-producing adenoma, and one subject with Cushing disease refractory to conventional treatment. Eleven sessions were performed for the nine patients. The patient data were reviewed for the clinical outcome and procedure-related complications over ten years.

**RESULTS:**

Patients with aldosterone-producing adenoma had clinical improvement: symptoms recurred in one case 96 months after ethanol ablation, and the other patient was still in remission 110 months later. All patients with pheochromocytoma had clinical improvement but were eventually submitted to surgery for complete remission. No significant clinical improvement was seen in patients with hypercortisolism due to primary macronodular adrenal hyperplasia or Cushing disease. Major complications were seen in five of the eleven procedures and included cardiovascular instability and myocardial infarction. Minor complications attributed to sedation were seen in two patients.

**CONCLUSION:**

Computed tomography-guided ethanol ablation does not appear to be suitable for the long-term treatment of hyperfunctioning adrenal disorders and is not without risks.

## INTRODUCTION

Laparoscopic adrenalectomy is the standard of care for hyperfunctioning benign adrenal disorders, such as pheochromocytomas and cortisol- or androgen-producing adenomas. It is also a therapeutic option for aldosterone-producing adenomas (APAs), adrenal hyperplasia resistant to medical treatment, and adrenocorticotropic hormone (ACTH)-dependent hypercortisolism, in which the source of ACTH production cannot be removed. Although considered safe, it is associated with an overall mortality of 0.2% and a mean length of stay of 3.3 days 1. Computed tomography (CT)-guided percutaneous instillation of sclerosing agents, such as absolute ethanol or acetic acid, has been used as an alternative treatment for adrenal lesions because these agents lead to coagulative necrosis 2–9. Potential advantages over laparoscopic surgery include lower complication rates, reduced length of stay and lower costs; however, few reports have been published to date, and none reported long-term follow-up data. The objective of this study was to evaluate the safety and efficacy of CT-guided percutaneous ethanol ablation (PEA) in a group of patients with hyperfunctioning benign adrenal disorders with long-term follow-up.

## MATERIALS AND METHODS

### Subjects

After institutional review board approval, we assessed our institutional clinical database. We retrospectively reviewed data from nine consecutive patients who underwent adrenal CT-PEA from March 2004 to July 2005. All patients were deemed unfit for surgery at that time. Written informed consent was obtained from all subjects. Seven patients (77.8%) were female. The mean age was 52.9 years (range, 38-71 years). Follow-up duration ranged from 1 to 110 months (Table 1).

The diagnoses of the patients were APA (2 cases), pheochromocytoma (3 cases), primary macronodular adrenal hyperplasia (PMAH, 3 cases – also known as ACTH-independent macronodular hyperplasia), and hypercortisolism due to Cushing disease refractory to conventional treatment (transsphenoidal hypophysectomy, pituitary radiotherapy and ketoconazole, 1 case). One of the patients with pheochromocytoma had an ACTH-producing pheochromocytoma (subject 6). All of the patients had biochemically proven adrenal hyperfunction before the procedure (elevated urinary metanephrines, high urinary cortisol levels and no suppression of serum cortisol after 1 mg dexamethasone test, or an aldosterone/plasma renin activity ratio >30 with hypokalemia and aldosterone >15 ng/mL). No adrenal venous sampling was performed.

Patients with primary aldosteronism and pheochromocytoma received spironolactone and oral alpha-1 adrenergic receptor antagonists, respectively, for at least one month before the procedure. Clinical improvement after treatment was evaluated by clinical signs and symptoms, reduction in the number or dosage of medications, and changes in laboratory examinations (endocrine markers, hormones and biochemistry) during the follow-up.

Data regarding one of the subjects (patient 6) have already been reported 10.

### Adrenal lesion characteristics

*Patients with APA (2) or pheochromocytoma (3)*: Each patient had only one nodule. Mean tumor size was 4.0 cm (range, 2.5-7.0 cm). Histopathological analysis was available from the 3 patients with a clinical diagnosis of pheochromocytoma: in one patient, percutaneous biopsy confirmed pheochromocytoma (subject 6); in the other two patients, ultimately performed adrenalectomy showed necrotic neoplasm (subject 7) and pheochromocytoma (subject 8).

*Patients with PMAH (3):* All had bilateral, enlarged macronodular adrenal glands. Histopathological analysis was available for two patients; in both cases, the histopathological results were consistent with the clinical diagnosis of PMAH: from percutaneous biopsy in subject 1 and from subsequent adrenalectomy in subject 3.

Patient with Cushing disease: Both adrenal glands were normal on CT.

### CT-PEA protocol

All of the procedures were performed by an experienced interventional radiologist (M.R.M). One of the patients underwent three PEA sessions; thus, there were a total of eleven procedures.

Seven of the procedures were performed using local anesthesia with lidocaine and conscious sedation with intravenous meperidine and midazolam, whereas four procedures were performed under general anesthesia.

Patients were placed in the prone position, and a 22-gauge Chiba needle was inserted into the adrenal target under CT guidance through a paravertebral access. Absolute ethanol laced with iodinated contrast medium was then instilled. CT images were obtained to confirm appropriate distribution of the ethanol. At the end of the procedure, a non-contrast-enhanced scan was performed to identify possible complications. When requested by the referring physician, biopsy was done at the beginning of the procedure.

All of the patients were treated with only one session, except for subject 1. The patients with PMAH had PEA performed in their largest nodules: subject 1 was treated unilaterally in the first session and bilaterally in the remaining procedures, subject 2 was treated bilaterally and subject 3 was treated unilaterally. The patient who had Cushing disease was treated bilaterally. All of the remaining patients, who had focal lesions, had PEA performed only in their single adrenal nodules. Therefore, eleven procedures were performed in nine patients. The ethanol volume instilled was calculated based on the nodule volume or adrenal volume: the mean volume of ethanol injected was 57.3 mL (range, 40.0-100.0 mL) per session or 39.4 mL per target ablated (range, 20.0-100.0 mL); relative to patient weight, the mean volume instilled was 0.8 mL/kg (range, 0.4-1.4 mL/kg).

All of the procedures were performed on an inpatient basis.

### ETHICS

Procedures were in accordance with the ethical standards of the Institutional Review Board and with the Helsinki Declaration of 1975, revised in 1983.

## RESULTS

### Clinical results

The two patients with APA had clinical improvement (improvement in hypokalemia and reduction in the dosage of anti-hypertensive medications). In one patient, PEA treatment was also associated with normalization of the aldosterone/plasma renin activity ratio, and she was still in remission 110 months after PEA. The other patient never had persistent normalization of the aldosterone/plasma renin activity ratio and had adrenalectomy for symptoms recurrence 96 months after ablation.

All of the patients with pheochromocytoma had improvement of clinical symptoms and laboratory examinations. In one of them, surgeons decided to perform adrenalectomy during a colectomy for colon adenocarcinoma one month after PEA despite clinical improvement after ablation; she died one year later from metastatic adenocarcinoma. The other two patients developed clinical recurrence and were subjected to laparoscopic adrenalectomy 39 and 83 months later.

One patient with PMAH developed clinical improvement and normalization of urinary cortisol and fasting glucose after two sessions of PEA, although ACTH remained suppressed. She recurred 10 months later and was retreated with PEA, again with the same results for another 68 months. She was then subjected to laparoscopic right adrenalectomy, which required conversion into open surgery and associated ipsilateral nephrectomy due to intraoperative renal bleeding. PEA failed in the other two patients with PMAH (one of them was lost to follow-up 7 months after treatment, and the other underwent adrenalectomy five months later).

The patient with Cushing disease did not show signs of improvement after PEA and was kept on medical treatment (Table 1).

### Complications

Complications were classified as minor or major events, according to the Society of Interventional Radiology's guidelines 11.

The review of medical data disclosed only immediate complications. Of the eleven procedures, five were associated with major complications, and two were associated with minor ones (Table 1). No procedure-related deaths were observed.

*Major complications:* One patient with APA developed symptomatic wide-QRS tachycardia and hypertension; this patient also developed neurologic symptoms approximately 72 hours after the procedure (ataxia, nystagmus and hemiparesis) and the symptoms resolved after 24 hours (brain imaging studies performed were normal). The three patients with pheochromocytoma developed severe intraprocedural hypertension (up to a mean value of 200 mmHg) after ethanol instillation that required intensive care for up to one day; one of the patients also had tachycardia with bigeminy requiring intravenous nitrates for several hours after the procedure for pressure control; and another patient had transitory hypotension afterward that needed vasoactive drugs. One patient, with Cushing disease, developed severe hypotension, central nervous system (CNS) depression and hypoxemia, and acute myocardial infarction, although coronary angiography performed the following day showed only left ventricular hypertrophy and normal coronary arteries.

Minor complications: Psychomotor alterations attributed to midazolam were seen in two patients, one of whom also developed short and self-limited hypoxia due to sedation.

## DISCUSSION

We presented here the long-term results of CT-PEA for the treatment of hormonally active adrenal lesions.

According to our results, PEA does not appear to be a reasonable alternative treatment to hormonally active adrenal lesions. Overall, the success rate was low (only one out of nine patients, 11.1%, was cured) and the incidence of complications was high (seven out of eleven procedures, 63.6%).

Other groups have reported treating functioning adrenal lesions with percutaneous chemical ablation (Table 2), for a total of 40 pheochromocytomas 5, 18 APAs 3,6–9 and 12 cortisol-producing adenomas (CPA) 6,7,9. Except for one patient who did not present a complete response to treatment 7 and another who recurred four years after ablation and was successfully retreated 8, both of them with APA, all other patients were considered to have been successfully treated. The only complications reported by these groups were pain 6,7,9, periprocedural increase in blood pressure 5,7,9 and adrenal insufficiency in one case 9. One patient, with a cortisol-producing adenoma, had severe pain and marked hypertension during the procedure, prompting its interruption, and had no complications when retreated during epidural anesthesia 7. No treatment was performed under general anesthesia by these groups.

Although technical differences, such as the ablating agent employed (acetic acid or ethanol), volume instilled, size of lesions, and number of treatment sessions performed, might play a role in explaining the seemingly better results reported in the literature, we believe that our longer follow-up data show that late recurrence may limit treatment in patients with PEA. Excluding the patient who underwent adrenalectomy one month after the ablation and who was clinically controlled, our patients who improved with PEA needed surgery 39, 68, 83 and 96 months after ablation, and only one was still cured after 110 months of follow-up; this pattern of late recurrence, also described by Chang et al 8, might not have been more often described due to the other published follow-ups ranging from 15 to 54 months.

PEA proved to be an even less effective procedure for the treatment of hypercortisolism secondary to nonfocal adrenal diseases, such as Cushing disease or macronodular hyperplasia. In our limited series, only one of the three patients with macronodular hyperplasia (subject 1) developed remission after ablation (lasting 10 and 68 months, after the second and third treatment sessions, respectively). The single patient with Cushing disease was also not cured after undergoing one session of bilateral adrenal PEA. Whether a greater number of ablation sessions or the use of acetic acid in these cases could have yielded better results remains unclear.

We observed significant cardiovascular side effects of PEA in several of these patients with adrenal disorders. All of the patients with pheochromocytoma received alpha-1 adrenergic receptor antagonists for at least one month before the procedure, as is typically done before adrenalectomy 12, although it did not prevent intraprocedural hypertension. Hypertensive crisis is a known complication of surgical treatment of this disorder 13. During adrenalectomy for pheochromocytoma, adrenal vein clamping prevents part of the catecholamines stored in the tumor from entering the blood circulation, and they are removed with tumor excision. Consequently, severe hypertension may occur during tumor manipulation in surgery but not after its completion; hypotension is the most common hemodynamic complication in the postoperative period 13. However, in adrenal PEA, catecholamines may be released during or after treatment, and hypertension might be observed several hours after the procedure. Additionally, similar to post adrenalectomy, we also observed postprocedural hypotension in one of our patients. Hypertensive crisis during adrenal PEA has not been previously reported. However, this complication, associated with a non-severe ventricular arrhythmia in one case 14, has been described in patients undergoing radiofrequency or cryoablation of nonfunctioning adrenal lesions 14–16 and metastatic pheochromocytomas and paragangliomas 17.

In patients without pheochromocytoma, cardiovascular effects included hypotension, CNS depression and hypoxemia, myocardial infarction, and tachyarrhythmia associated with hypertension. Hypotension and CNS depression and hypoxemia observed in subject 4 could have resulted from the systemic effects of ethanol 18, as was also reported in one subject treated by Minowada et al 19; this may have been potentiated by prior benzodiazepine and opiate administration. Hypotension has also been observed in image-guided PEA for treating other organs, such as hepatocellular carcinoma in the liver and several cases of cardiovascular collapse have been reported 20,21. Acute myocardial infarction followed by an unremarkable coronary angiography may have been related to an increased myocardial demand secondary to left myocardial hypertrophy and a lower oxygen supply as a result of hypotension and transitory hypoxemia; transient elevation of pulmonary artery pressure and myocardial depression may also result from ethanol spilling into the systemic circulation 22. Retrospectively, another diagnostic possibility (which has more recently been reported after adrenal cryoablation (15)) would have been the apical ballooning syndrome: it corresponds to reversible cardiomyopathy precipitated by stress that presents with clinical features of myocardial infarction, with elevation of cardiac enzymes, and temporary left ventricular dyskinesia without obstructive coronary disease 23. Cardiac arrhythmias may be related to both hyperaldosteronism 24 and ethanol 25.

The complications of adrenal PEA herein reported prompted us to change from doing the procedure under sedation to under general anesthesia. Patients with pheochromocytoma who undergo PEA, similar to those who undergo adrenalectomy 13, might need admittance to an intensive care unit due to hemodynamic instability. General anesthesia, mechanical ventilation and invasive blood pressure monitoring may be important for performing adrenal PEA safely.

The ideal volume of ethanol administered in PEA is unclear. Although prior studies of sclerotherapy of vascular anomalies with ethanol suggest that volumes greater than 1.0 mL/kg are associated with acute morbidity or toxicity 22, most of our patients were treated with smaller volumes and still developed complications. The low ethanol volumes used in Xiao's study 6 might be safer, although a higher number of sessions may be required. Wang et al 5 treated forty benign pheochromocytomas after intraprocedural phentolamine and reported no hypertensive crisis; the volume of ethanol instilled in their case was reported to be “equivalent to size of the tumor”.

More recently, image-guided radiofrequency ablation (RFA) has also been successfully used for treating benign functioning adrenal lesions 26–31. Whereas the ethanol distribution within tumors is limited by septa and can be unpredictable, RFA creates a relatively homogeneous ablation zone. It has already been shown to be more effective than PEA for treating hepatocellular carcinoma (HCC) 32,33. Analysis of the pooled results from these cohorts 26–31 is very promising: a total of 55 lesions have been treated (44 APA, 7 CPA, 3 pheochromocytomas and 1 sex steroid-producing tumor), with biochemical success in 51 patients and clinical success in 53 of them. Further studies should compare RFA to laparoscopic adrenalectomy, the current gold standard for hyperfunctioning adrenal lesions.

Our study has some limitations. It included a small sample size and heterogeneous population. Its retrospective design limits the availability of patient data.

In summary, our results indicate that ethanol ablation of benign adrenal lesions involves significant risks and lacks long-term efficacy.

## AUTHOR CONTRIBUTIONS

Frenk NE and Sebastianes F were responsible for data collection and analysis, and manuscript writing. Lerario AM and Fragoso MC were responsible for the project development and manuscript editing. Mendonca BB was responsible for the project development and manuscript editing. Menezes MR was responsible for the project development, data analysis and manuscript editing.

## Figures and Tables

**Table 1:** Patient Data.

Pt	Age (y) and Gender	Diagnosis	Lesion treated	Ethanol volume in ml (ethanol volume per weight in ml/kg)	Anesthesia	Complications	Clinical results
1	51 F	PMAH	Left macronodular adrenal gland	40 (0.6)	Conscious sedation	None	Failure
1	51 F	PMAH	Right and left macronodular adrenal glands	R: 20 / L: 30 (0.8)	Conscious sedation	None	Clinical improvement; recurrence and repeated PEA 10 months later
1	52 F	PMAH	Right and left macronodular adrenal glands	R: 20 / L: 70 (1.4)	General anesthesia	None	Clinical improvement; recurrence and adrenalectomy 68 months later
2	45 F	PMAH	Right and left macronodular adrenal glands	R: 35 / L: 25 (0.8)	Conscious sedation	None	Failure, lost to follow-up
3	65 F	PMAH	Left macronodular adrenal gland	40 (0.4)	Conscious sedation	Minor: Transient drowsiness, bradypnea and hypoxemia (midazolam)	Failure, adrenalectomy 5 months later
4	38 F	Cushing disease	Right and left adrenal glands	R: 40 / L: 40 (1.0)	Conscious sedation	Major: Hypotension, CNS depression and MI	Failure
5	50 F	APA	3.0 cm nodule in the left adrenal gland	50 (1.0)	Conscious sedation	Minor: Agitation, short and self-limited hypoxia (sedation)	Clinical improvement for 110 months
6	71 M	Pheo	4.5 cm nodule in the left adrenal gland	40 (0.7)	Conscious sedation	Major: Hypertension followed by hypotension	Clinical improvement; recurrence and adrenalectomy 83 months later
7	56 F	Pheo	7.0 cm nodule in the right adrenal gland	100 (1.4)	General anesthesia	Major: Hypertension and tachyarrhythmia, admitted to ICU	Clinical improvement, adrenalectomy 1 month later (synchronous to colectomy)
8	61 F	Pheo	3.0 cm nodule in the left adrenal gland	40 (0.6)	General anesthesia	Major: Hypertension and tachycardia, admitted to ICU	Clinical improvement; recurrence and adrenalectomy 39 months later
9	39 M	APA	2.5 cm nodule in the left adrenal gland	40 (0.5)	General anesthesia	Major: Symptomatic wide-QRS tachycardia and hypertension; transient neurological symptoms	Clinical improvement; recurrence and adrenalectomy 96 months later

*Pt* = Patient, *M* = Male, *F* = Female, *PMAH =* Primary Macronodular Adrenal Hyperplasia, *APA =* Aldosterone-producing adenoma, *Pheo* = Pheochromocytoma, *R* = Right; *L* = Left; *CNS* = Central nervous system; *MI* = Myocardial infarction; *ICU =* Intensive care unit.

**Table 2:** Published Results on Percutaneous Chemical Ablation of Pheochromocytomas, Aldosterone-Producing and Cortisol-Producing Adenomas of the Adrenal Gland.

	Pheo	APA	CPA	Size: min-max (mean) (cm)	Agent	Repeated sessions?	Follow-up (mo)
Rossi et al. 3	-	1	-	2.0	Ethanol	No	17
Liang et al. 9	-	2	1	1.3-3.5 (2.3)	Acetic acid	No	15-18
Wang et al. 5	40	-	-	1.1-4.1 (NR)	Ethanol	No	23-54
Minowada et al. 7	-	5	5	1.0-3.8 (2.2)	Acetic acid^a^	Yes	5-69
Xiao et al. 6	-	9	6	1.9-4.4 (2.8)	Ethanol/Acetic acid^b^	Yes	24
Chang et al. 8	-	1	-	1.0	Ethanol	Yes^c^	>48^c^
Our results	3	2	-	2.5-7.0 (4.0)	Ethanol	No	1-110

*Pheo* = Pheochromocytomas, *APA =* Aldosterone-producing adenomas, *CPA =* Cortisol-producing adenomas, *NR* = Not reported, *Acetic acid^a^* = one patient was submitted to one session of ethanol ablation, *Ethanol/Acetic acid^b^* = Ethanol was used for tumors up to 3.0 cm and acetic acid for larger lesions; *Yes^c^; >48^c^* = patient successfully retreated 4 years after first PEA with another ablation session, but subsequent follow-up time not disclosed.
